# A Chalcone Synthase-like Bacterial Protein Catalyzes Heterocyclic C-Ring Cleavage of Naringenin to Alter Bioactivity Against Nuclear Receptors in Colonic Epithelial Cells

**DOI:** 10.3390/metabo15030146

**Published:** 2025-02-21

**Authors:** Ebru Ece Gülşan, Farrhin Nowshad, Meredith Davis Leigh, Jimmy Walter Crott, Hyejin Park, Greg Martin, Stephen Safe, Robert S. Chapkin, Arul Jayaraman, Kyongbum Lee

**Affiliations:** 1 Department of Chemical and Biological Engineering, Tufts University, Medford, MA 02155, USA; ecegulsan@gmail.com; 2 Artie McFerrin Department of Chemical Engineering, Texas A&M University, College Station, TX 77843, USA; fnowshad@tamu.edu; 3 Jean Mayer USDA Human Nutrition Research Center on Aging, Tufts University, Boston, MA 02111, USA; meredith.davis685@duke.edu; 4 Department of Pathology and Laboratory Medicine, Boston University School of Medicine, Boston, MA 02118, USA; jcrott@bu.edu; 5 Department of Veterinary Physiology and Pharmacology, Texas A&M University, College Station, TX 77843, USA; hpark@cvm.tamu.edu (H.P.); gmartin@cvm.tamu.edu (G.M.); ssafe@cvm.tamu.edu (S.S.); 6 Department of Nutrition, Texas A&M University, College Station, TX 77843, USA; r-chapkin@tamu.edu

**Keywords:** flavonoids, naringenin, ring cleavage, gut microbiome, aryl hydrocarbon receptor

## Abstract

Gut microbial metabolism of dietary flavonoids leads to a diverse array of bioactive products that are closely associated with human health. Combining enzyme promiscuity prediction, metabolomics, and in vitro model systems, we identified a chalcone-synthase-like bacterial polyketide synthase that can initiate the metabolism of naringenin by catalyzing the C-ring cleavage. This was validated using a mutant strain of the model organism *Bacillus subtilis* (ATCC 23857). Our prediction–validation methodology could be used to systematically characterize the products of gut bacterial flavonoid metabolism and identify the responsible enzymes and species. In vitro experiments with Caco-2 cells revealed that naringenin and its bacterial metabolites differentially engage the aryl hydrocarbon receptor (AhR) and orphan nuclear receptor 4A (NR4A). These results suggest that metabolism by gut bacterial species could directly impact the profile of bioactive flavonoids and influence inflammatory responses in the intestine. These results are significant for understanding gut-microbiota-dependent physiological effects of dietary flavonoids.

## 1. Introduction

Flavonoids are the largest class of naturally occurring polyphenolic phytochemicals, numbering over 6000 structurally distinct molecules [1]. As dietary compounds, they are particularly abundant in plant-based foods such as fruits and vegetables that are considered health-promoting. The health benefits of flavonoids are generally associated with their antioxidant and anti-inflammatory activities [2], although the potency varies widely even among flavonoids of the same subclass. There is no clear consensus regarding the molecular mechanisms underlying the bioactivities of flavonoids. In vitro studies have shown that flavonoids can engage specific cellular receptors [3], but it remains to be established whether and which of the pathways regulated by these receptors are responsible for the flavonoids’ beneficial effects in vivo. Further, it is unclear whether the flavonoids directly bind the receptors or are first transformed in the body into metabolic products which then activate the receptors.

In the intestine, potential molecular targets for flavonoids are the aryl hydrocarbon receptor (AhR) and nuclear receptor 4A (NR4A). Both are transcription factors capable of binding a variety of metabolite ligands to regulate inflammatory pathways in colonic epithelial cells [4,5]. Studies have shown that flavonoids can exhibit agonist or antagonist activities to induce or inhibit the expression of AhR and NR4A target genes [6,7]. These two receptors can also bind phenolic acids derived from flavonoids [8,9]. In contrast to many flavonoid compounds, phenolic acids are readily absorbed in the intestine and are typically found at higher plasma concentrations. For example, the average daily plasma concentrations of quercetin and naringenin in humans are on the order of 1 µM [10], whereas their major phenolic acid metabolites phenylacetic acid and phenylpropionic acid are present at hundred-fold or higher concentrations [11]. This raises the intriguing question of whether flavonoid-derived metabolites are quantitatively important ligands for the AhR, NR4A, and other flavonoid relevant receptors.

Upon ingestion, flavonoids are only partially absorbed and therefore remain available for metabolism by commensal microorganisms residing in the intestine or gut microbiota. Most dietary flavonoids (except for flavan-3-ols) are ingested in their glycoside form. The first step of their metabolism is deglycosylation. For *O*-coupled flavonoids, deglycosylation can be catalyzed by human glycosidases such as lactase–phlorizin hydrolase and cystolic β-glycosidase [12]. *C*-glycosides, however, are quantitatively metabolized by gut bacteria [13]. Following glycoside hydrolysis, the resulting aglycones can be absorbed and conjugated into glucuronide and sulfate forms in the intestine or liver by phase II enzymes. Alternatively, they can be further metabolized by gut bacteria. Gut bacteria can perform a variety of reactions, including ring cleavage, de/methylation, and di/hydroxylation, to generate metabolites that cannot be formed through host metabolism alone. In addition to providing an energy source, metabolism of flavonoids also serves as a detoxification mechanism for some gut bacterial species [14]. Addition or removal of hydroxy and/or methoxy groups has been observed under anaerobic conditions at all three flavonoid rings [15,16]. Cleavage of a C-O bond, however, has only been reported for the central C-ring. Once a flavonoid has undergone C-ring cleavage, it can be further degraded into short-chain fatty acids (SCFAs) (from the A-ring) and hydroxyphenyl acids (from the B-ring).

Not all gut bacteria can catalyze degradation of flavonoids into these metabolites. For example, only a handful of species have been identified that can perform *O*-demethylation of isoflavones and flavanones [15,17,18]. Moreover, the enzymatic pathways of flavonoid metabolism in gut bacteria are largely unresolved. Flavonoids are not natural substrates of gut bacterial enzymes. Consequently, reactions of flavonoid metabolism have been attributed to more general classes of enzymes [19]. Modifications such as hydrolysis, de/methylation, and di/hydroxylation have been linked to glycosyltransferases, methyltransferases, and oxidoreductases. However, the specific enzymes that belong to these classes and the bacterial species carrying the enzymes remain to be elucidated.

In vitro studies using human fecal cultures found that *Eubacterium ramulus* and *Flavonifractor plautii* can break down certain flavonoids, including quercetin, luteolin, and naringenin, into SCFAs and phenolic acids [20,21]. A more recent study by Braune et al. purified and characterized the enzyme catalyzing the C-ring contraction of (+)-taxifolin to form alphitonin in *E. ramulus* and showed that the gene encoding this enzyme had complete sequence identity to the gene encoding chalcone isomerase (CHI) [22]. This study also identified another flavanonol-isomerizing enzyme in *F. plautii* based on the enzyme’s 50% sequence identity to the CHI from *E. ramulus*. Another study by Braune et al. demonstrated that *E. ramulus* harbors an oxygen-sensitive NADH-dependent reductase that can open the heterocyclic C-ring of several flavanones and flavanonols (naringenin, eriodictyol, liquiritigenin, and homoeriodictyol) [23]. In *F. plautii*, Yang et al. identified a flavone reductase that catalyzes the hydrogenation of the C2-C3 double bond of flavones and flavonols to initiate C-ring opening [24]. Miyake et al. showed that anaerobically grown *Clostridium butyricum* can cleave the C3-C4 bond in the C-ring of eriodictyol to form phenolic acids [25]. Pereira-Caro et al. co-incubated *Bifidobacterium longum* and *Lactobacillus rhamnosus* with orange juice flavanones under anaerobic conditions and identified several phenolic acids as metabolic products [26]. These studies demonstrate that selected gut bacteria are capable of degrading flavonoids by opening the C-ring; however, the responsible enzymes are largely unknown.

In this study, we investigate the metabolism of a model flavonoid, naringenin, into its C-ring cleavage product, 3-(4-hydroxyphenyl) propionic acid (3,4-HPPA), by gut bacterial enzymes. Using metabolic modeling, we predict that this metabolism proceeds through a chalcone-synthase-like bacterial polyketide synthase and confirm this prediction using in vitro culture experiments. Additionally, we explore the interaction of naringenin and its bacterial metabolites with the aryl hydrocarbon receptor (AhR) and nuclear receptor 4A (NR4A) in intestinal epithelial cells to better understand their potential physiological impact. Through this work, we aim to establish a systematic framework for characterizing flavonoid metabolism by gut bacteria and identifying the responsible enzymes and species.

## 2. Materials and Methods

### 2.1. Cell Lines, Bacterial Strains, Culture Conditions, and Reagents

The young adult mouse colonic (YAMC) cell line was originally obtained from the R. H. Whitehead Ludwig Cancer Institute (Melbourne, Australia). The cells were maintained in RPMI 1640 medium with 5% fetal bovine serum (FBS), 5 units/mL mouse interferon-γ (IF005) (EMD Millipore, Burlington, MA, USA), and 0.1% ITS “−” minus (insulin, transferrin, selenium) (41-400-045) (Life Technologies, Grand Island, NY, USA) at 33 °C in the presence of 5% CO_2_. Caco-2 human colon cancer cells were obtained from the American Type Culture Collection (ATCC, Manassas, VA, USA) and maintained in Dulbecco’s modified Eagle’s medium (DMEM) nutrient mixture supplemented with 20% (*v*/*v*) FBS, 1% (*v*/*v*) 100× MEM nonessential amino acid solution (Gibco), and 1% (*v*/*v*) 100× penicillin–streptomycin (Pen–Strep) solution (Sigma-Aldrich, St. Louis, MO, USA) at 37 °C in the presence of 5% CO_2_. TCDD (99%) used in this study was synthesized in the Safe laboratory. Unless otherwise noted, all other chemicals used in the study were purchased from Sigma-Aldrich (St. Louis, MO, USA). *B. subtilis* ATCC 23857, *F. plautii* ATCC 49531, *L. plantarum* ATCC 14917, and *E. coli* ATCC BAA-1430 were obtained from ATCC (Manassas, VA, USA). *P. lactis* DSM 28626 was obtained from the German Collection of Microorganisms and Cell Cultures (DSMZ, Braunschweig, Germany). Mutant *B. subtilis* BKK22050 (trpC2 ΔbcsA::kan) was obtained from the Bacillus Genetic Stock Center (BGSC, Columbus, OH, USA). *B. subtilis 168* (BGSC accession number: 1A1; ATCC catalog number 23857) was used as the wild-type strain for the construction of the mutant strain (accession number: P54157.BCSA_BACSU). Both wild-type (ATCC 23857) and mutant (BKK22050) strains of *B. subtilis* were cultured in nutrient broth supplemented with potato extract (Sigma-Aldrich, St. Louis, MO, USA). *L. plantarum* was cultured in MRS broth (Sigma-Aldrich, St. Louis, MO, USA). *P. lactis* was cultured in Luria–Bertani (LB) broth (Sigma-Aldrich, St. Louis, MO, USA). *E. coli* and *F. plautii* were cultured in brain–heart infusion (BHI) broth (Becton, Dickinson and Company, Franklin Lakes, NJ, USA) supplemented with 0.5% yeast extract (Becton, Dickinson and Company, Franklin Lakes, NJ, USA), 0.05% cysteine (Alfa Aesar, Tewksbury, MA, USA), 0.05% hemin, and 0.02% vitamin K (Sigma-Aldrich, St. Louis, MO, USA).

### 2.2. Bacterial Monoculture

The *B. subtilis* strains were cultured aerobically in nutrient broth as described above. Cultures grown to an OD (absorbance at 600 nm) of 1.0 were diluted 10-fold and treated with varying doses of naringenin (10 or 100 μM; stock solutions were prepared using DMSO as solvent and then filter-sterilized) or vehicle (0.1% *v*/*v* DMSO). Following the naringenin or vehicle treatment, *B. subtilis* cultures were incubated at 37 °C for 72 h. *P. lactis*, *L. plantarum*, *E. coli*, and *F. plautii* cultures were grown in their respective media as mentioned above inside an anaerobic chamber with 85% N_2_, 10% CO_2_, and 5% H_2_ (Coy Lab, Grass Lake, MI, USA) and treated with naringenin (10, 100, or 1000 μM) or vehicle in a similar manner. Each monoculture experiment was run in triplicate. Samples were collected for metabolite extraction by sacrificing the cultures after 24, 48, and 72 h of incubation.

### 2.3. Facal Culture

Fresh fecal material was collected from 14-week-old female C57BL/6J mice fed ad libitum a standard chow diet (Teklad Rodent Diet 8604, Envigo Bioproducts, Madison, WI, USA). The fecal pellets were immediately transported to an anaerobic chamber with 85% N_2_, 10% CO_2_, and 5% H_2_ (Coy Lab, Grass Lake, MI, USA) using anaerobic transport medium (BD Diagnostics, Franklin Lakes, NJ, USA). All reagents were introduced into the anaerobic chamber a day before the experiment to ensure that they were pre-reduced by the start of the experiment. Anoxic phosphate-buffered saline (PBS) (10 mL) containing 0.5 g/L of cysteine was added to 1 g of fecal sample. The suspension was homogenized by vigorous shaking and vortexing. The samples were left for 15 min to allow large particles to settle. To culture fecal bacteria, gut microbiota medium (GMM) was used, which was prepared as described previously [27]. Sterile conical tubes containing 9 mL of GMM were inoculated with 300 µL of the fecal suspension. The inoculated tubes were then treated with varying doses of naringenin (10 or 100 µM) or vehicle (0.1% *v*/*v* DMSO) at the time of inoculation (day 0). The fecal cultures were then incubated under anaerobic conditions at 37 °C and sacrificed for metabolite extraction after 6, 12, 24, and 48 h of incubation. For the ^13^C isotopic labeling experiment, the fecal cultures were treated with 100 μM of phloretin-(hydroxyphenyl-^13^C_6_) at the time of inoculation and incubated anaerobically as described above. Based on the observation that the concentration of ^13^C_6_-3,4-HPPA at 6 and 12 h was below the limit of quantification, the cultures were incubated for 24, 48, and 72 h and then sacrificed for metabolite extraction.

### 2.4. Metabolite Extraction

Metabolites were extracted from the harvested cell culture samples using a solvent-based method. Briefly, 50 µL of culture sample was mixed with 200 µL of ice-cold methanol using a vortex mixer and kept on ice for 60 min. The sample–solvent mixture was then centrifuged at 15,000× *g* at 4 °C for 5 min. The supernatant was filtered through a 0.22 µm sterile nylon filter (Corning). The filtrate was then passed through a 3 kDa molecular weight cutoff filter (Amicon Ultra, Sigma-Aldrich, St. Louis, MO, USA) placed in a collection tube by centrifuging the sample tube at 15,000× *g* for 45 min at 4 °C. The filtrate from the collection tube was then mixed with 2 mL of sterile water, lyophilized to a pellet using a freeze dryer (FreeZone, Labconco Corporation, Kansas City, MO, USA), and stored at −80 °C until further analysis. Prior to LC-MS/MS analysis, the freeze-dried samples were reconstituted in 250 µL of 50% (*v*/*v*) methanol/water.

### 2.5. Targeted Analysis of Naringenin Metabolites

The analytical method for detecting naringenin, phloretin, and 3,4-HPPA was developed in-house and validated for reliability. The samples were analyzed using product ion scan experiments performed on a triple-quadrupole time-of-flight instrument (5600+, AB Sciex) coupled to a binary pump high-performance liquid chromatography (HPLC) system (1260 Infinity, Agilent, Santa Clara, CA, USA). Chromatographic separation was achieved on a Synergi 4 µm Hydro, 250 mm × 2 mm, 80 A reverse-phase column (Phenomenex, Torrance, CA, USA) maintained at 30 °C using a solvent gradient method. Solvent A was 0.2% (*v*/*v*) formic acid in water, and solvent B was 100% acetonitrile. The gradient method was as follows: 0–30 min—95% A to 45% A; 30–38 min—45% A to 95% A. The flow rate was set to 0.6 mL/min. The injection volume was 10 µL. Raw data were processed in PeakView 2.1 to determine the ion peaks. The peak areas in the extracted ion chromatograms were manually integrated using MultiQuant 2.1 (AB Sciex). The identities of detected compounds were confirmed by matching their retention time and/or MS/MS spectra to high-purity standards run on the same instrument using the same method. Limit of detection (LoD) values were determined as follows: naringenin, 15.22 µM; 3,4-HPPA, 2.33 µM; and phloretin, 2.1 µM. Calibration curves were established using two-fold serial dilutions (naringenin, 0.12–62.5 µM; 3,4-HPPA, 0.31–500 µM; and phloretin 3.9–1000 µM), with each concentration injected in duplicate. System suitability was assessed through six serial injections of the same standard, where the relative standard deviation (RSD) for retention time and peak area remained below 0.5%, demonstrating method robustness. The following high-purity standards were used: naringenin (≥95% purity, Sigma-Aldrich), naringenin chalcone (≥95.0% purity, HPLC, Sigma-Aldrich), phloretin (≥99% purity, HPLC, Sigma-Aldrich), and 3-(4-hydroxyphenyl) propionic acid (98% purity, Sigma-Aldrich).

### 2.6. AhR Activity Assay

Caco-2 and YAMC cells were maintained in their respective media as described above. Cells were seeded at 60–70% density and were sub-confluent after treatment and subsequent analysis. YAMC cells (1.2 × 10^7^ cells) were treated with TCDD (10 nM) and/or compounds such as naringenin (50–100 µM) and naringenin chalcone (10–200 µM) for 2 or 24 h. Caco-2 cells (5 × 10^6^ cells) were treated with TCDD and/or compounds for 2 h. For co-treatment, Caco-2 cells were treated with 100 µM of 3,4-HPPA as well as 5 mM butyrate (Thermo Fisher Scientific, Inc., Waltham, MA, USA) for 24 h. The cells were then fixed with 1% formaldehyde, and the cross-linking reaction was stopped by addition of 0.125 M glycine. After washing with phosphate-buffered saline, the cells were scraped and pelleted.

### 2.7. NR4A1/2 Binding Assay

Binding affinities of naringenin and 3,4-HPPA for NR4A1 and NR4A2 were assayed based on quenching of tryptophan fluorescence as previously described [28]. The ligand binding domains (LBDs) of NR4A1 and NR4A2 were incubated at a fixed concentration (0.5 μM) in PBS with different concentrations of naringenin and 3,4-HPPA. Fluorescence spectra were obtained at 285/330 nm (slit width: 5 nm).

### 2.8. Quantitative Real-Time Reverse Transcriptase PCR

Total RNA was extracted using an RNA isolation kit from the cells according to the manufacturer’s protocol. cDNA synthesis was performed from the total RNA of cells using the High-Capacity RNA-to-cDNA Kit (Applied Biosystems, Foster City, CA, USA). Real-time PCR was carried out using Bio-Rad SYSR Universal premix for 1 min at 95 °C for initial denaturing, followed by 40 cycles of 95 °C for 15 s and 60 °C for 1 min in the Bio-Rad iCycler (MyiQ2) real-time PCR system. Gene expression values were analyzed using the comparative CT method and normalized to expression levels of TATA-binding protein (TBP). The sequences of the primers used for real-time PCR are shown in Table 1.

### 2.9. Cytokine Measurement

Caco-2 cells were cultured in DMEM supplemented with 10% (*v*/*v*) FBS, 2 mM glutamine, and 1% penicillin–streptomycin (Life Technologies, Grand Island, NY, USA). Sub-confluent cultures were maintained in a humidified incubator at 37 °C with 5% CO_2_. Cells were seeded in a 96-well plate at a density of 1.5 × 10^4^ cells per well and allowed to attach overnight. The next day, the growth medium was replaced with serum-free medium. The cells were then treated with naringenin, 3,4-HPPA, or vehicle for 2 h followed by addition of *IL-1β* (50 ng/mL). After 24 h, the supernatants were collected and clarified using centrifugation at 1500 RPM and 4 °C for 10 min. Secretion of *IL-8* was measured in the cell-free supernatant samples using the Human *IL-8/CXCL8* Quantikine ELISA Kit, following the manufacturer’s protocol (R&D Systems, Minneapolis, MN, USA, D8000C).

### 2.10. Murine Intestinal Microbiota Model

To define the atom group transformation operators used for predicting flavonoid metabolism, we assembled a model of metabolic reactions catalyzed by enzymes in representative strains of murine intestinal microbiota. We previously compiled a list of organisms to represent bacteria detected in anaerobic batch culture of cecal contents from 6- to 8-week-old female C57BL/6J mice [29]. We compared this list against the Mouse Intestinal Bacterial Collection (miBC) [30] and removed strains belonging to genera absent in the miBC. The rationale for this step was to obtain a reduced list of organisms that are not specific to the experimental system used in the previous study and thus more broadly representative of culturable murine intestinal bacteria. We then added miBC strains absent from the reduced list but belonging to a genus represented in the list. These steps yielded a total of 106 strains belonging to 18 genera. These strains were processed as described previously [29] to obtain a matrix of organisms and their enzymes’ EC numbers.

### 2.11. Model Prediction of Flavonoid Metabolism

To predict products of flavonoid metabolism and gut bacterial enzymes responsible for these products, we modified a scheme previously developed (PROXIMAL) to analyze the products of xenobiotic transformation reactions catalyzed by liver cytochrome P450 enzymes [31]. In this scheme, the reactant–product pair(s) (RPAIR) of an enzymatic reaction [32] are analyzed to identify changes in the reactant’s atom group(s) that transform the reactant into the product. These changes define the enzyme’s transformation pattern that is associated with a reaction center and its first- and second-level neighboring atoms. If a substrate of interest has an atom group that matches this pattern for an enzyme, then a transformation operator associated with the enzyme is applied to generate a product from the substrate. In the present study, we performed the RPAIR analysis on enzymes in the above-described murine intestinal microbiota model. A total of 5932 unique atom group transformation operators were identified for the model. These operators were applied to each of the 19 flavonoid compounds to predict their bacterial metabolites. To allow for two-step transformations analogous to phase I and II reactions for xenobiotic transformation, the products from one round of metabolite predictions were used as inputs for a second round. The metabolites from both rounds were filtered to eliminate trivial products such as simple sugars, CO_2_, and water. We also eliminated compounds that are not cataloged as metabolites in KEGG. Predictions were evaluated against experimental reports of gut bacterial metabolism. Notably, for flavonoids with documented gut bacterial metabolism, our model predictions were largely consistent with experimental evidence. For instance, 12 of the 16 predicted naringenin metabolites were detected in cultures of individual gut bacteria or fecal material incubated with naringenin [33,34,35,36].

### 2.12. Prediction of Naringenin C-Ring Cleavage Enzymes

Enzymes in the microbiota model corresponding to operators that generate C-ring cleavage metabolites for naringenin were assembled into a list with their corresponding RPAIRs. These RPAIRs were clustered using a binning method based on atom-pair descriptors generated from their SMILES identifiers in ChemMine Tools [37]. The similarity of RPAIRs was calculated as the Tanimoto coefficient of corresponding atom-pair descriptors. Distances between clusters were determined by single linkage. The RPAIRs clustered with naringenin were then visually inspected. If the reaction described by an RPAIR resembled the predicted transformation (i.e., reaction resulted in aromatic ring cleavage), the corresponding enzyme(s) were marked as having potential to catalyze the transformation with naringenin as a substrate. Each reaction was assumed to be reversible unless otherwise specified. Predicted key enzymes were matched with the organism by using the organism–enzyme matrix from the murine cecal microbiota model. Enzyme promiscuity analysis was also evaluated against two other computational tools for predicting small molecule metabolism, BioTransformer [38] and Way2Drug [39], for further validation.

## 3. Results

### 3.1. Enzyme Promiscuity-Based Model Links Gut Microbiota Composition and Potential for Metabolizing Structurally Diverse Flavonoids

We adapted a tool for prediction of promiscuous enzyme activity on xenobiotic chemicals (PROXIMAL) to investigate possible products of gut bacterial flavonoid metabolism. Previously, we assembled a model of metabolic reactions catalyzed by enzymes of bacteria detected in anaerobic batch culture of fecal material from 6- to 8-week-old female C57BL/6J mice. For the present study, we modified this model to predict promiscuous enzyme activities in murine intestinal microbiota. The previous metabolic model was pared down to a subset of representative strains cataloged in the Mouse Intestinal Bacterial Collection (miBC). This yielded a total of 106 strains belonging to 18 genera. These strains were then searched against the KEGG GENOME database and UniProtKB to identify the enzymes encoded in their genomes and match these enzymes with corresponding Enzyme Commission (EC) numbers. The reactant–product (RCLASS) data associated with these EC numbers were analyzed using PROXIMAL to determine atom group transformations in the enzymes’ natural substrates. These atom group transformations were used to define operators for an enzyme that, when applied to potential nonnatural substrates having atom groups that match the enzyme’s natural substrate, predict the corresponding reaction products (Figure S1). In total, the murine intestinal microbiota model enzymes mapped to approximately 12,000 unique atom group transformation operators.

These operators were applied to 19 dietary flavonoids and their glucosides belonging to five major subclasses found in fruits and vegetables (Table S1). Cellular metabolism of exogenous chemicals often occurs in two steps, with the first and second steps resulting in substrate activation and metabolism, respectively. To mimic this process, the products predicted by the operators for each of the 19 flavonoids were used as substrates for a second round of predictions. Combined, the two rounds predicted 1427 metabolites having unique KEGG compound identifiers. We did not attempt an exhaustive search of the published literature for these compounds, as the lack of reported findings regarding gut bacterial metabolism of a flavonoid is not evidence that metabolism does not occur. When gut bacterial metabolism was reported for one of the 19 flavonoids, the experimental evidence largely agreed with our predictions.

**Figure 1:**
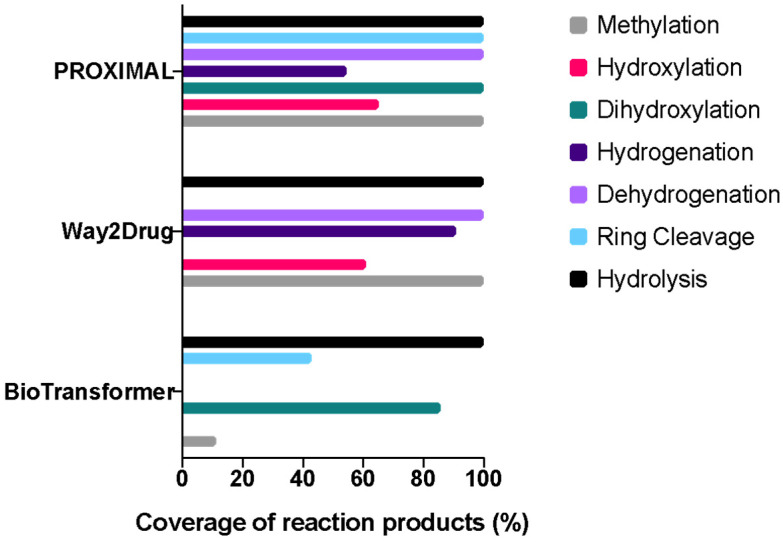
Comparison of reaction products predicted by PROXIMAL, Way2Drug, and BioTransformer. Five flavonoids were selected to represent the following subclasses: flavonol (quercetin), flavanone (naringenin), flavone (apigenin and luteolin), and isoflavone (genistein). Coverage was calculated with respect to the total number of distinct reaction products collectively predicted by the three tools. Full (100%) coverage by a tool for a reaction type indicates that the tool predicted all metabolites predicted by the other two tools for the same reaction type.

We next evaluated our enzyme promiscuity analysis against two other computational tools for predicting small molecule metabolism, BioTransformer and Way2Drug. This evaluation focused on five flavonoids representing the major subclasses flavonol (quercetin), flavanone (naringenin), flavone (apigenin and luteolin), and isoflavone (genistein). The evaluation also included naringin, the glucoside conjugate of naringenin. The predictions were compared for products of methylation, hydroxylation, dihydroxylation, hydrogenation, dehydrogenation, C-ring cleavage, and hydrolysis. Overall, our enzyme-promiscuity-based approach (PROXIMAL) generated the most comprehensive coverage of reaction products, except for hydrogenation products (Figure 1). Way2Drug predicted the largest number of hydrogenation products but the fewest dihydroxylation and C-ring cleavage products. PROXIMAL and Way2Drug predicted the same set of methylation and dehydrogenation products, with BioTransformer predicting fewer products. All three tools predicted the same set of hydrolysis products.

### 3.2. Gut Bacterial Metabolism of Flavonoids Is Predicted to Vary by Molecular Structure and Taxa

**Figure 2:**
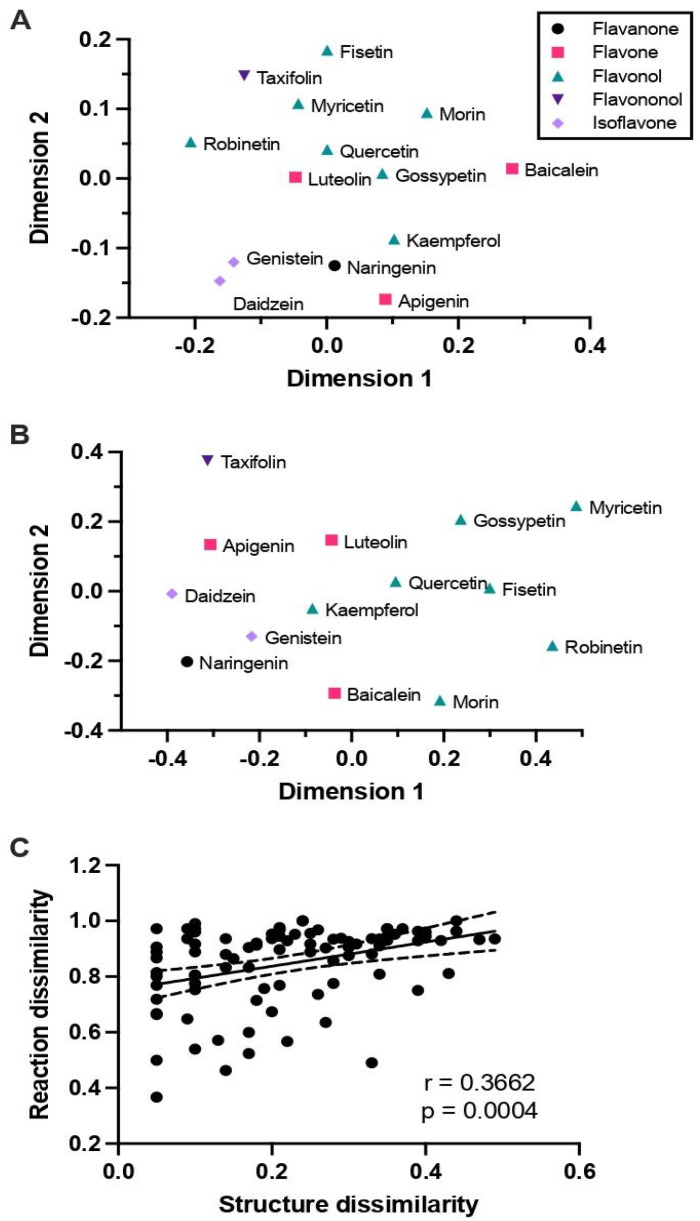
Structural similarity and predicted reaction similarity of flavonoids. (**A**) Multidimensional scaling (MDS) map for 15 flavonoid aglycones, where the compounds’ coordinates were assigned based on a matrix of relative pairwise distances representing structural dissimilarities calculated using the SIMCOMP2 tool. Symbols and colors indicate the compounds’ subclasses. (**B**) MDS map where the compounds’ coordinates were assigned based on their predicted reaction patterns. (**C**) Correlation between structural and reaction dissimilarities of flavonoids. Solid and dashed lines show the best fit linear regression model and 95% confidence intervals, respectively.

We investigated the impact of flavonoid structure on the predicted reactions. Structural similarity scores were calculated for 15 aglycones using the SIMCOMP2 tool [40] and structural similarity and predicted reaction similarity of flavonoids are shown in Figure 2.

Figure 2A shows a multidimensional scaling (MDS) map where the distance between a pair of compounds corresponds to the dissimilarity (1—similarity score) between the pairs. Overall, flavonoids from the same subclass were structurally more alike than compounds from other subclasses and grouped closer together on the MDS plot. For example, almost all the flavonols (fisetin, myricetin, morin, robinetin, quercetin, and gossypetin) were grouped together in the top half of the plot except for kaempferol. The isoflavones (genistein and daidzein) formed a tight pair in the bottom of the plot. However, the flavones did not show this grouping; luteolin and baicalein were grouped with the flavonols, whereas apigenin was grouped closer to the isoflavones. These trends indicated that the subclass of a flavonoid only partially predicts the structural similarity to other flavonoids. We observed a similar trend when we constructed an MDS map based on the flavonoids’ similarity of predicted reactions (Figure 2B). The isoflavones and flavonols formed two groups on the left and right side of the plot, respectively, whereas the flavones did not show this grouping. A correlation analysis confirmed that there was a modest but significant association between the structural and reaction dissimilarities of flavonoids (Figure 2C). Based on these results, we chose quercetin, luteolin, naringenin, and genistein for further analysis to represent flavonoids of different subclasses with varying structures and predicted reaction profiles. We also included apigenin, which belongs to the same subclass as luteolin but was grouped more closely with the isoflavones and naringenin, a flavanone.

**Figure 3:**
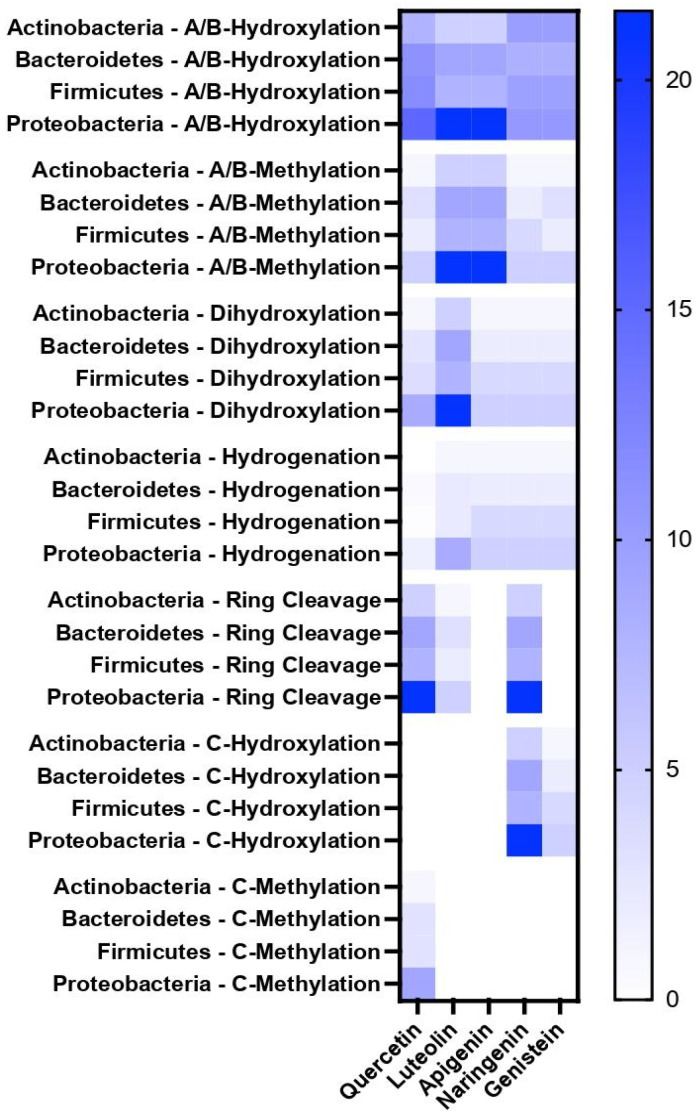
Distribution of predicted flavonoid metabolizing enzymes across different bacterial phyla. The color scale corresponds to the number of matching enzymes in the phylum. The number of matches for a phylum was normalized by the number of strains included in the model for the phylum. Each row corresponds to a different combination of phylum and type of reaction.

We next investigated the distribution of predicted flavonoid metabolizing enzymes among the model’s bacterial groups. We tabulated the enzymes corresponding to the PROXIMAL operators for predicted metabolites of the selected flavonoids and searched for matching enzymes in the microbiota model. The matches were determined based on the first three digits of the enzymes’ EC numbers. We did not use the fourth digit, which specifies the natural substrate or cofactor of an enzyme. A central premise of our predictions is that enzymes can catalyze reactions of non-natural substrates. We focused on methylation, hydroxylation, dihydroxylation, hydrogenation, and C-ring cleavage as these reactions were most frequently predicted for the selected flavonoids. To investigate whether the functional group position matters, we separately tabulated hydroxylation and methylation reactions on A/B and C rings. Figure 3 shows the predicted distribution of flavonoid metabolizing enzymes across different bacterial groups at the phylum level. The trends shown in the heatmap were similar at lower taxonomic levels, as genera and families belonging to the same phylum had similar sets of enzymes predicted to metabolize flavonoids (Table S2). Hydroxylation and methylation on the A and B rings were predicted for all phyla and flavonoids. Naringenin and genistein had the largest number of enzymes predicted to catalyze A or B ring hydroxylation, while apigenin and luteolin had the most matches for A or B ring methylation. Dihydroxylation followed a similar trend, with luteolin having the largest number of matching enzymes. Hydrogenation was predicted to occur less commonly for all flavonoids, with Actinobacteria lacking the enzymes altogether. Unlike A and B ring reactions, C ring reactions were predicted for only some of the flavonoids. Hydroxylation was predicted mainly for naringenin, whereas methylation was predicted only for quercetin. C-ring cleavage showed a heterogenous distribution across flavonoids and phyla. While we did not predict any enzymes that could catalyze this reaction for apigenin and genistein, we predicted multiple such enzymes for quercetin and naringenin in strains from all four phyla.

### 3.3. Monoculture Experiments Confirm Naringenin Metabolism by Selected Gut Bacteria

**Figure 4:**
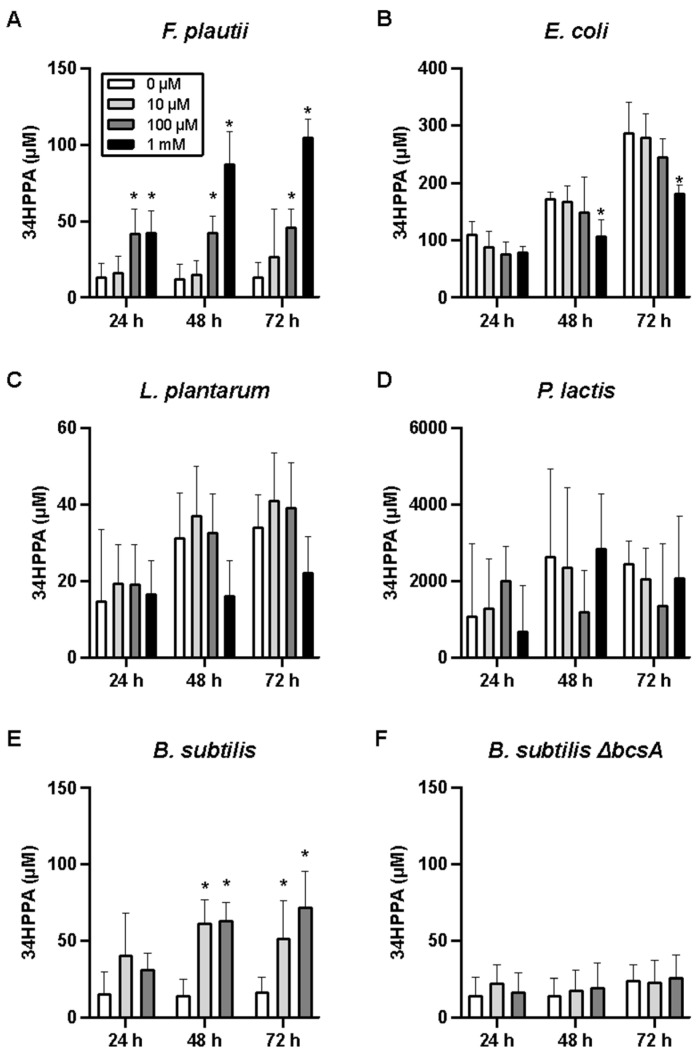
Concentrations of 3-(4-hydroxyphenyl) propionic acid (3,4-HPPA) in naringenin-treated bacterial monocultures. (**A**) *F. plautii*, (**B**) *E. coli*, (**C**) *P. lactis*, (**D**) *L. plantarum*, (**E**) wild-type *B. subtilis*, and (**F**) mutant *B. subtilis* lacking chalcone synthase (Δ*bcsA*). Data shown are means ± SD (N = 3 biological replicates). Asterisks (*) indicate a significant difference (*p* < 0.05) compared to the vehicle control (0 µM naringenin) at the corresponding time point.

Enzymatic cleavage of a C-O bond in the C-ring of flavanones has been extensively studied, yet *F. plautii* is the only species detected in murine intestine reported to catalyze this reaction. The results in Figure 4 suggested that there are other murine gut bacteria that can catalyze this reaction. To experimentally validate this prediction, we cultured selected species from the microbiota model in the presence of naringenin and analyzed the spent medium and cell pellets for accumulation of 3,4-HPPA, a metabolite that results from C-ring cleavage of the flavanone. We observed a dose- and time-dependent increase in 3,4-HPPA when anaerobically grown *F. plautii* were treated with naringenin (Figure 4A), consistent with previously reported experiments [20,41]. We observed a time-dependent increase in 3,4-HPPA when naringenin was added to the culture of *E. coli*, but this accumulation occurred independently of naringenin dose (Figure 4B). This indicated that *E. coli* can produce 3,4-HPPA but does not use naringenin as a substrate. The amount of 3,4-HPPA detected in the *E. coli* culture was significantly higher than *F. plautii*. This is likely due to the higher growth rate of *E. coli*, as the amounts are comparable for the two cultures when normalized to the culture OD. Neither the *P. lactis* nor the *L. plantarum* culture showed a dose- and time-dependent accumulation of 3,4-HPPA upon naringenin treatment (Figure 4C,D). These results are consistent with our prediction that species in these two genera cannot catalyze naringenin C-ring cleavage.

We next investigated the enzyme(s) that could be responsible for naringenin C-ring cleavage. In many plants, flavanone C-ring cleavage is catalyzed by chalcone isomerase (CHI). However, none of the strains in the microbiota model has a gene for CHI or a CHI ortholog. We further examined the PROXIMAL results by clustering the predicted flavonoid reactions based on their RCLASS similarity. This analysis found that a chalcone synthase (CHS)-like enzyme could accept naringenin as a substrate to form naringenin chalcone. We then created a phylogenetic tree with all bacterial strains in the microbiota model that have genes homologous to CHS from *Medicago sativa* (a legume expressing CHS with strong naringenin binding activity) with a sequence similarity score > 80%. This analysis found that *B. subtilis* has a bacterial polyketide synthase (*bcsA*) with a high degree of sequence similarity to *M. sativa* CHS.

To investigate whether this enzyme catalyzed naringenin C-ring cleavage in *B. subtilis*, we incubated wild-type *B. subtilis* with varying concentrations of naringenin (10 or 100 µM) and observed a time-dependent increase in 3,4-HPPA (Figure 4E); this increase was not observed when the cells were exposed to only the vehicle. We performed the same dose–response experiment using a mutant strain, *B. subtilis* BKK22050, which lacks *bcsA*. Unlike the wild-type strain, the mutant strain did not show a dose- and time-dependent increase in 3,4-HPPA (Figure 4F), indicating that CHS activity is necessary for naringenin C-ring cleavage in *B. subtilis*.

### 3.4. Fecal Microbiota Metabolizes Naringenin Through C-Ring Cleavage

**Figure 5:**
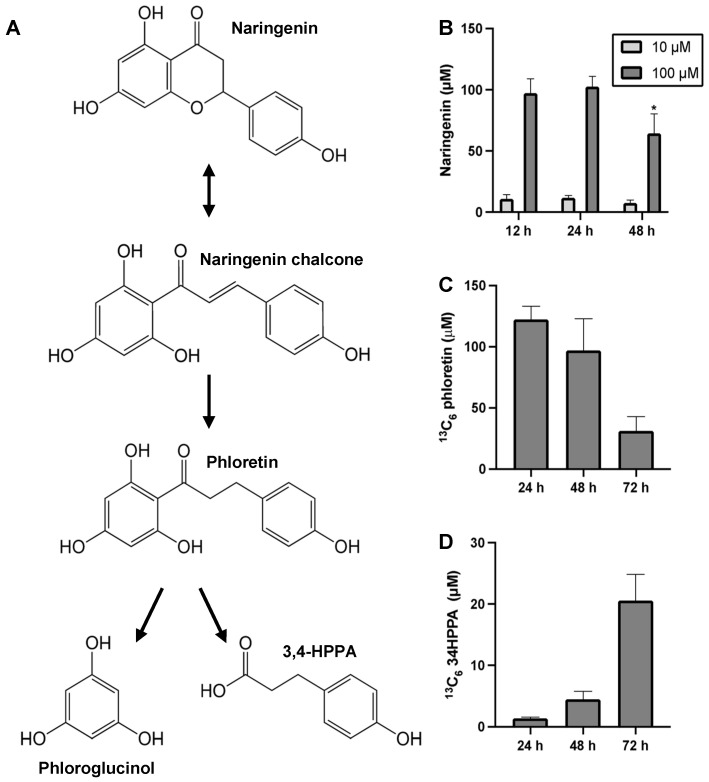
Conversion of naringenin to phloretin and phloretin-(hydroxyphenyl-13C6) to 13C6-3,4-HPPA in fecal culture. (**A**) Proposed pathway for naringenin metabolism via C-ring cleavage in the fecal culture. The dotted arrow shows direct conversion of naringenin to phloretin via a flavanone-cleaving reduction of the C-ring. (**B**) Concentration of naringenin in the fecal culture at different times after naringenin supplementation. An asterisk (*) indicates a significant difference compared to the initial timepoint at 12 h. (*p* < 0.05). (**C**) Phloretin-(hydroxyphenyl-13C6) and (**D**) 13C6-3,4-HPPA concentrations at different times after 100 µM phloretin-(hydroxyphenyl-13C6) supplementation. Data shown are means ± SD (N = 3 biological replicates). An asterisk (*) indicates a significant difference (*p* < 0.05) compared to the vehicle control (0 µM) at the corresponding time point.

To investigate naringenin degradation in a mixed community of gut bacteria, we incubated murine fecal cultures with varying doses of naringenin (Figure 5A). At the highest dose (100 µM), we observed a significant ~40% decrease in naringenin after 48 h of incubation, indicating net utilization of naringenin (Figure 5B); however, we did not detect naringenin chalcone. Although high concentrations of 3,4-HPPA were detected in the culture, this was not dependent on naringenin dose. One possible explanation for this is that the fecal culture microorganisms produced 3,4-HPPA from other sources, as we observed for *E. coli* (Figure 4B). We also detected phloretin, which lies upstream of 3,4-HPPA in the C-ring cleavage pathway of naringenin, but the concentrations detected in the fecal culture were below the quantification limit of our LC-MS assay (~10 µM).

To determine if conversion of phloretin to 3,4-HPPA occurred in the fecal culture, we supplemented the culture medium with ^13^C_6_-phloretin (without naringenin) and monitored the accumulation of ^13^C_6_-3,4-HPPA. We observed a time-dependent decrease in ^13^C_6_-phloretin (Figure 5C) and a concomitant increase in ^13^C_6_-3,4-HPPA (Figure 5D). We also detected high concentrations of unlabeled 3,4-HPPA, consistent with the monoculture observations that murine gut bacteria can produce 3,4-HPPA not only through flavonoid metabolism but also through other pathways.

### 3.5. Naringenin and Its Metabolites Elicit Different Biological Activities

**Figure 6:**
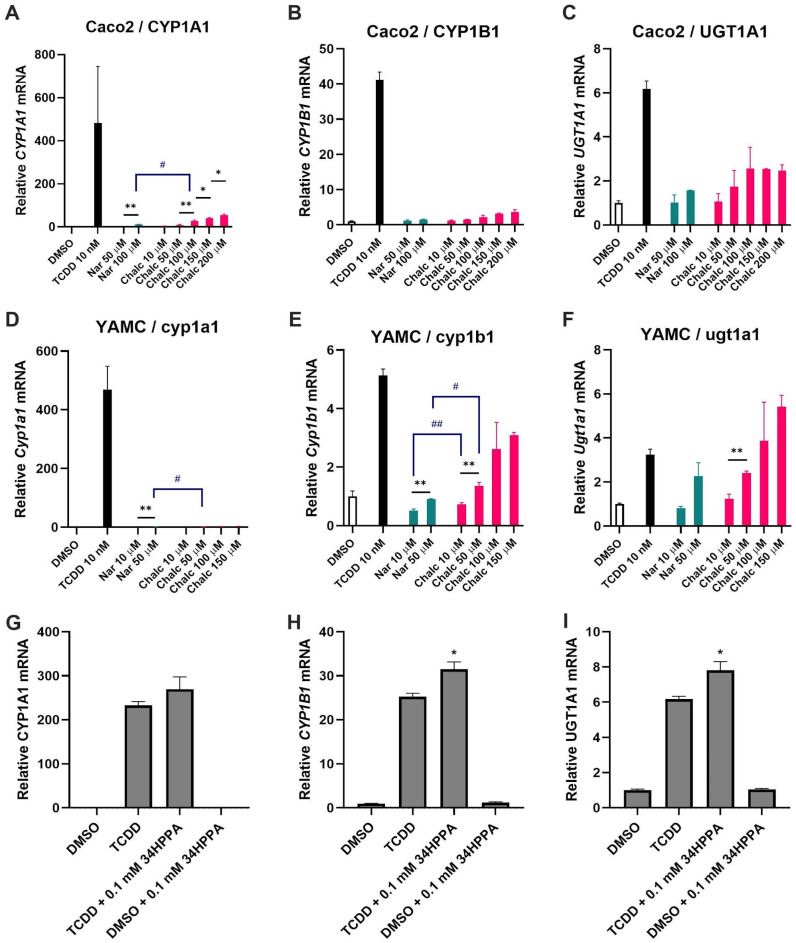
Induction of AhR-responsive genes by naringenin (Nar) and naringenin chalcone (Chalc). (**A**) CYP1A1, (**B**) CYP1B1, and (**C**) UGT1A1 in Caco-2 cells. (**D**) Cyp1a1, (**E**) Cyp1b1, and (**F**) Ugt1a1 in YAMC cells. (**G**–**I**) Induction of CYP1A1, CYP1B1, and UGT1A1 in Caco-2 cells by 3,4-HPPA alone and in combination with 10 nM 2,3,7,8-tetrachlorodibenzo-p-dioxin (TCDD). The cells were treated with varying concentrations of the indicated compounds for 24 h. Gene (mRNA) expression was determined by real-time PCR. The data shown are means ± SD (N = 3 biological replicates). For panels (**A**–**F**), asterisks indicate a significant difference (*, *p* < 0.05; **, *p* < 0.01) compared to vehicle control (DMSO), whereas hashes (#) indicate a significant difference (#, *p* < 0.05; ##, *p* < 0.01) between naringenin and naringenin chalcone at the same concentration. For panels G–I, an asterisk (*) indicates a significant difference (*p* < 0.05) compared to TCDD, which was used as the positive control for AhR-dependent induction of gene expression.

Having confirmed that naringenin can be metabolized by gut bacteria to 3,4-HPPA by way of naringenin chalcone and phloretin, we next investigated whether the parent flavanone and its metabolic products elicit different biological responses. We have previously shown that many flavonoids exhibit AhR ligand activity [3,6,42]. Therefore, we assayed the induction of AhR-responsive *CYP1A1, CYP1B1*, and *UGT1A1* gene expression in murine (YAMC) as well as human (Caco-2) colonic epithelial cell models (Figure 6).

In Caco-2 cells, both naringenin and naringenin chalcone dose-dependently induced the expression of *CYP1A1* (Figure 6A) and *CYP1B1* (Figure 6B). The level of *CYP1A1* induction was stronger for the chalcone compared to naringenin (27-fold vs. 11-fold relative to vehicle control, DMSO, at 100 μM); however, neither compound showed strong induction compared to the positive control (10 nM TCDD). Previously, we found that naringenin significantly reduced cell viability at concentrations greater than 100 μM [42]. Interestingly, the chalcone was far less cytotoxic, and we were able to test concentrations up to 200 μM. Both naringenin and its chalcone induced a significant, dose-dependent increase in *UGT1A1* expression that was of the same order magnitude as TCDD (Figure 6C). In YAMC cells, we observed dose-dependent induction of *Cyp1a1* (Figure 6D), *Cyp1b1* (Figure 6E), and *Ugt1a1* expression (Figure 6F) by naringenin chalcone, with the induction levels of *Cyp1b1* and *Ugt1a1* reaching a similar magnitude to the positive control. In part because of the dose limitation imposed by naringenin’s cytotoxicity at higher concentrations, we observed negligible (*Cyp1a1*) or insignificant (*Cyp1b1* and *Ugt1a1*) induction by naringenin.

One of the factors influencing AhR ligand activation in colonic epithelial cells is crosstalk with other small molecule modulators of gene expression. Recent studies showed that short-chain fatty acids (SCFAs) such as acetate, butyrate, and propionate can enhance TCDD-induced Ah-responsive gene expression in colonic epithelial cells [42]. We therefore investigated whether 3,4-HPPA, which, unlike the chalcone intermediate, did not induce AhR target gene expression, could influence gene expression in conjunction with an agonist. In Caco-2 cells, 3,4-HPPA significantly enhanced *UGT1A1* and *CYP1B1* expression when combined with TCDD (Figure 6G–I). This is qualitatively similar to the synergistic effect of butyrate on AhR-responsive *CYP1A1*, *CYP1B1,* and *UGT1A1* gene expression previously observed in these cells [43].

**Figure 7:**
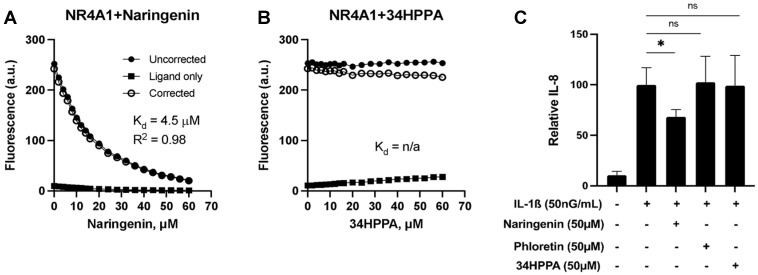
Loss of naringenin ligand binding and immunomodulatory activity upon metabolism to 3,4-HPPA. Concentration-dependent quenching of tryptophan fluorescence in the ligand-binding domain (LBD) of NR4A1 with (**A**) naringenin and (**B**) 3,4-HPPA. (**C**) IL-8 concentration in conditioned medium following IL-1β stimulation of Caco-2 cells treated with naringenin, 3,4-HPPA, and phloretin. The data shown in panel (C) are means ± SD (N = 3 biological replicates). An asterisk (*) indicates a significant difference (*p* < 0.05) compared to the positive control.

There is increasing evidence that the nuclear receptor 4A subfamily, in addition to the AhR, could also play a role in mediating the biological activities of flavonoids. Recent in vitro studies have demonstrated that kaempferol and quercetin can bind the ligand binding domain (LBD) of NR4A1 to inhibit pro-oncogenic NR4A1-regulated pathways [28]. We investigated whether naringenin or its gut bacterial metabolites can also bind nuclear receptor 4A LBDs (Figure 7). Direct binding assays using LBD of NR4A1 showed a strong binding affinity of naringenin with a K_D_ of 4.5 μM, whereas 3,4-HPPA did not show any binding activity (Figure 7A,B). Finally, having found that naringenin and its microbial metabolic products elicit different biological responses as AhR activators and NR4A1/2 ligands, we next investigated their immunomodulatory effects in colonic epithelial cells by stimulating Caco-2 cells with interleukin-1β *(IL-1β*) and measuring the secretion of *IL-8*, a neutrophil chemotactic factor. Whereas naringenin dose-dependently decreased *IL-8* secretion, neither 3,4-HPPA nor phloretin had a significant effect (Figure 7C).

## 4. Discussion

In this work, we utilized a prediction tool for enzyme promiscuity to investigate gut bacterial metabolism of dietary flavonoids. We identified a novel bacterial enzyme, *BcsA,* that can catalyze the C-ring cleavage reaction of a flavanone and validated the enzyme’s role in metabolizing naringenin to 3,4-HPPA in an in vitro culture of *B. subtilis*. We further showed that a naringenin degradation pathway that proceeds through C-ring cleavage is active in anaerobic batch culture of murine fecal microbiota. We also showed that intermediates of naringenin’s C-ring cleavage degradation pathway elicit different biological activities such as AhR and NR4A1/2 ligands and mediate differential immunomodulatory responses in intestinal epithelial cell lines.

A survey of published in vitro and in vivo studies found strong corroborating evidence for the predicted metabolites. Among 16 predicted naringenin metabolites, 12 were detected in cultures of individual gut bacteria or fecal material incubated with naringenin [34,35,36,37]. Burapan et al. reported methylation of luteolin and apigenin by *Blautia* sp. MRG-PMF1 under anaerobic conditions that match the predictions of the present study [44]. Isorhamnetin and tamarixetin were detected in rat plasma as methylated metabolites of quercetin [45,46], consistent with our prediction results. Using fecal slurry cultures from human donors, Di Pede et al. found that quercetin degrades into various phenylpropanoic, benzoic, and phenylacetic acid derivatives [47]. These studies attributed the observed metabolites to C-ring cleavage of quercetin, consistent with the broad availability of this reaction as predicted by our enzyme promiscuity model (Figure 3).

Not all predictions could be corroborated with reported findings. For example, Soukup et al. [48] and Lee et al. [49] suggested that the first step of anaerobic microbial degradation of genistein is hydrogenation on C-ring, a reaction our model did not predict. Further, we did not find evidence in the literature that genistein undergoes any of the other predicted reactions such as de/methylation and di/hydroxylation. It is possible that certain flavonoid-derived metabolites may not be readily measured in vitro due to challenges in culturing the microorganisms that express the responsible enzymes. Additionally, the resulting metabolites may be intermediates that are further degraded into smaller organic acids. Another reason some metabolites were not observed by previous studies could be that these metabolites were not in the scope of the studies’ targeted analyses. In this regard, computational predictions of promiscuous enzyme activity could complement experimental investigations to systematically characterize the metabolism of flavonoids, which are exogenous to gut bacteria.

There have been limited attempts to develop prediction methods for gut microbial metabolism of dietary phytochemicals, whereas there are several well-known approaches for predicting the metabolism of chemicals in the environment or human tissue, such as enviPath [50] and Meteor Nexus [51]. These approaches have typically used rule-based methods to generate biotransformation pathways, where the rules are derived from a knowledgebase of enzymatic reactions. BioTransformer is a recently developed tool that combines rule-based and machine learning approaches to generate biotransformation rules [38]. Notably, this tool includes a module for gut microbial metabolism built from databases of metabolites detected in bodily fluids after consumption of polyphenols [52] and other phytochemicals. One key difference between our method and BioTransformer is that the latter assigns many of its rules for the gut microbial metabolism to unspecified generic enzymes, whereas the present study links each biotransformation operator to one or more specific gut microbial enzymes. On the other hand, rule-based methods such as BioTransformer can predict multi-step transformations. In principle, the PROXIMAL algorithm could be used for this purpose by processing the outputs from one iteration as inputs for the next iterations. However, this would be impractical, as the algorithm lacks a reasoning engine to select reactions that are more likely to occur. Future implementations of PROXIMAL could incorporate learning-based approaches based on retrosynthesis, where a heuristic search algorithm (e.g., Monte Carlo Tree Search) is used to explore the biotransformation space [53].

In addition to BioTransformer, we evaluated our predictions against Way2Drug RA, which identifies the reacting atoms of drug-like compounds undergoing biotransformation by cytochrome P450 (CYP) enzymes and transferases. The reaction database for this tool also includes microbial flavonoid reactions such as aromatic hydroxylation and *O*-glucuronidation. This database, however, cannot be customized by the user to model a specific microbiome. Compared to both BioTransformer and Way2Drug RA, the enzyme promiscuity analysis performed in the present study predicted a more diverse set of flavonoid metabolites, while also modeling a specific set of enzymes in a user-defined microbiome. The latter feature is critical for investigating which gut bacteria are responsible for flavonoid metabolism. In the present study, we successfully validated a novel prediction that *B. subtilis* can metabolize flavanone via C-ring cleavage.

Naringenin is a flavanone abundant in citrus fruits. The starter unit for naringenin biosynthesis is 4-coumaroyl-CoA, which is derived from either phenylalanine or tyrosine depending on the plant [54,55]. Chalcone synthase (CHS) combines 4-coumaroyl-CoA with three malonyl-CoA molecules to produce naringenin chalcone, which is then isomerized to naringenin by chalcone isomerase (CHI) [56]. This reaction is reversible, and CHI can also cleave the C-ring of naringenin to produce naringenin chalcone (Figure S2). Zeng et al. studied the fermentation of naringin, the glycoside form of naringenin, in anaerobically cultured microbiota from rat fecal material and found that *p*-coumaric acid was one of the metabolic products [57]. In the present study, we did not detect any enrichment of *p*-coumaric acid in naringenin-treated fecal cultures. The study by Zeng et al. [57] used rat intestinal and cecal material for their anaerobic fermentation experiments, whereas our study used murine fecal material. These fecal materials from two different host animals likely differ in the composition and abundance of microbial species, which could explain why we did not detect *p*-coumaric acid in our fecal culture experiments.

Gut bacterial degradation of flavonoids results in the formation of various organic acids, notably hydroxylated phenyl propionic acids. Although it is well-established that this degradation proceeds through cleavage of the heterocyclic C-ring [58], the species and enzymes carrying out this metabolism remain largely unknown. Recent studies have identified a CHI-like enzyme and NADH-dependent reductase in *E. ramulus* and a flavone reductase in *F. plautii* as gut bacterial enzymes capable of opening the C-ring of naringenin [23,24]. These studies also suggested that phloretin hydrolase is responsible for further degradation of flavonoid-derived chalcones into phenolic acids. In the present study, we identified a bacterial CHS-like enzyme that can catalyze C-ring cleavage of the flavanone naringenin. Enzymes of the CHS superfamily share high similarity in their amino acid sequence, structure, and general catalytic principles and contain the conserved Cys–His–Asn catalytic triad at the binding site [56]. Although 4-coumaroyl-CoA is the natural substrate for plant CHS, earlier studies have shown that CHS of *Medicago sativa* can also use naringenin as a substrate [59]. In vitro monocultures of *F. plautii* and *B. subtilis* treated with naringenin showed a dose- and time-dependent accumulation of 3,4-HPPA, whereas *E. coli* and other gut bacteria predicted to lack the CHS-like enzyme did not show this trend. However, we did not detect other naringenin ring cleavage pathway intermediates such as naringenin chalcone and phloretin in the *F. plautii* and *B. subtilis* monocultures. This could be due to the rapid metabolism of these intermediates into 3,4-HPPA. We also did not detect phloroglucinol, another product of the ring cleavage pathway, which can be completely degraded by intestinal microbiota into acetate, butyrate, and CO_2_ [60].

Unlike the monocultures of *F. plautii* and wild-type *B. subtilis*, we did not detect a dose-dependent increase in 3,4-HPPA concentration in the fecal cultures upon naringenin addition. While 3,4-HPPA is a major product of naringenin metabolism by the gut bacteria [35], the fecal culture contains other sources for producing this phenolic acid. Aromatic amino acids such as phenylalanine and tyrosine, which are present in the culture medium (GMM), are also precursors for 3,4-HPPA. This could explain the presence of the higher concentration of 3,4-HPPA in fecal cultures independent of naringenin dosage and incubation time. The conversion of substrates other than naringenin, for example, via Stickland fermentation [61], could also explain an apparent excess of 3,4-HPPA formation in the wild-type *B. subtilis* cultures (Figure 4E, 10 μM M condition). Stickland fermentation of tyrosine uses *p*-coumaroyl-CoA as an intermediate, which could be upregulated in the presence of naringenin chalcone (Figure S2). A more specific intermediate of naringenin metabolism via C-ring cleavage is naringenin chalcone. Like *F. plautii* and *B. subtilis* monocultures, we did not detect the chalcone intermediate. In addition to rapid conversion of the chalcone intermediate to phloretin, another possible explanation is that naringenin was directly converted to phloretin as reported previously by Braun et al. [23] (Figure 5A). Phloretin was detected at elevated levels in naringenin-treated fecal cultures relative to vehicle-treated controls. However, the detected phloretin concentrations were below quantifiable levels. To trace phloretin’s metabolic fate, we supplemented the culture medium with ^13^C_6_-phloretin instead of naringenin. We observed a time-dependent decrease in ^13^C_6_-phloretin concentration and a concomitant increase in ^13^C_6_-3,4-HPPA. We did not detect any unlabeled phloretin in the culture, which indicates that the phloretin detected in the naringenin treatment experiment is derived from naringenin rather than any other medium component. Taken together, these results suggest that phloretin derived from naringenin is metabolized to 3,4-HPPA in the fecal culture. However, the conversion of phloretin to 3,4-HPPA was relatively low. Whereas the labeled phloretin concentration decreased to ~30 µM by 72 h, labeled 3,4-HPPA increased to only ~20 µM (Figure 5C,D). It is possible that 3,4-HPPA was further degraded to other phenolic acids, e.g., 4-hydroxyphenyl acetic acid, 3-phenyl propionic acid, and benzoic acid. Another possibility is that phloretin was converted to phloridzin (phloretin 2′-*O*-glucose), which can be formed by bacterial phloretin-2′-*O*-glycosyltransferase (P2′GT) in the presence of glucose in bacteria culture media [62]. 

Recent structure activity relationship experiments on flavonoids in host cells indicated that there is a significant difference in AhR ligand activity between the parent compounds and their gut bacterial metabolites [63,64]. For example, quercetin is a weak agonist for AhR in T47D breast cancer cells [65]. In the same cell line, 3,4-dihydroxyphenylacetic acid (DOPAC), a microbial metabolite reported to derive from quercetin [8], did not affect AhR activation [9]. It is worth noting that reported results on products of flavonoid metabolism appear to vary with different model systems. Another study on quercetin metabolism in an in vitro mixed-culture model of human colonic microbiota did not detect DOPAC. Instead, this study reported 3-hydroxyphenylacetic acid and 3-(3-hydroxyphenyl)-propionic acid as the major quercetin metabolites [66]. Tamura et al. studied quercetin metabolism by fecal microbiota from healthy elderly human subjects and found that *Fusobacteriaceae* and *Enterobacteriaceae* affected quercetin bioavailability by inhibiting its degradation by other bacteria. They also reported that the abundance of *Sutterellaceae* and *Enterobacteriaceae* is positively correlated with quercetin degradation, suggesting that the fate of quercetin depends on microbiota composition [67].

In this study, we characterized naringenin and its ring cleavage metabolites as AhR ligands in Caco-2 and YAMC cells. Naringenin chalcone had moderate AhR activity, whereas naringenin and 3,4-HPPA had little to no activity. We also found that YAMC cells were more responsive than Caco-2 cells to induction of AhR-mediated gene expression by naringenin chalcone. Our results agree with other studies on ligand activation of the AhR that the cell type plays a significant role in target gene expression [68]. Another factor influencing AhR ligand activation is crosstalk with other gene expression modulators. Studies have shown that short-chain fatty acids (SCFAs) enhance the responsiveness of the AhR to structurally diverse ligands due, in part, to their role as histone deacetylase inhibitors [44]. Park et al. reported that acetate, butyrate, and propionate enhance TCDD-induced *CYP1A1* expression in Caco-2 cells [42]. In the present study, we found that 3,4-HPPA exhibited SCFA-like synergy with an AhR agonist, significantly enhancing TCDD-induced *CYP1A1* and *CYP1B1* expressions even though the naringenin metabolite by itself did not show AhR ligand activity. These results suggest that gut bacterial metabolism of dietary flavonoids could influence AhR-mediated responses in the intestine not only by shaping the profile of AhR active flavonoid ligands but also by producing other small molecules that interact with AhR pathways. While we demonstrated that 3,4-HPPA is the major metabolite of naringenin degradation via C-ring cleavage, the fecal culture environment contains aromatic amino acids such as phenylalanine or tyrosine that are also precursors for 3,4-HPPA. Therefore, the 3,4-HPPA concentration in the intestine is likely high even when flavonoids are only partially degraded. Our results also suggest naringenin chalcone is an AhR-active ligand. Given that naringenin, naringenin chalcone, and 3,4-HPPA are likely to occur together in the intestine, it is important to consider the potential for synergistic activation of the AhR by 3,4-HPPA, naringenin chalcone, and other microbially derived AhR ligands. To the best of our knowledge, this is the first study to investigate the synergistic induction of AhR-regulated gene expression by a microbially derived flavonoid metabolite.

NR4A is a family of orphan nuclear receptors that plays a significant role in innate and adaptive immunity, maintaining cellular homeostasis and pathophysiology. In many diseases, cellular stressors induce various NR4A members. For example, NR4A1 levels increase in many solid tumor-derived cancer cells with enhanced metabolic rates [69]. In this study, we showed that naringenin binds with high affinity to NR4A1 and NR4A2, whereas its metabolite 3,4-HPPA was inactive. In effect, gut microbial metabolism of naringenin leads to the inactivation of an NR4A1/2-active compound. Similarly, naringenin dose-dependently decreased *IL-1ß* and induced *IL-8* production in Caco-2 cells, whereas phloretin and 3,4-HPPA had no effect. Hamers et al. reported that overexpression of NR4A1 contributes to the anti-inflammatory response of Caco-2 cells by decreasing the expression of *IL-8* [70]. This is consistent with the findings from the present study, which show that the NR4A1/2-active compound naringenin inhibits *IL-8* expression in Caco-2 cells, while 3,4-HPPA is NR4A1/2-inactive and does not elicit the anti-inflammatory effect.

In summary, this study establishes a generalizable prediction–validation framework for characterizing gut microbial flavonoid metabolism and identifying key enzymes using naringenin as a model flavonoid. We provided novel evidence that a CHS-like bacterial polyketide synthase catalyzes the heterocyclic C-ring cleavage of naringenin, with our model predicting its presence in only a subset of gut bacteria, such as *Bacillus*, potentially explaining inter-individual differences in flavonoid metabolism. We also demonstrated that a flavonoid and its metabolites elicit different biological activities such as AhR and NR4A1/2 ligands, which could explain their differential effects in mediating inflammatory responses in intestinal epithelial cells. While the focus of our study was on developing and testing the prediction framework, in vivo studies with naringenin are needed to further validate our findings. Future studies can build on this work by exploring microbial community dynamics and underlying metabolic interactions with flavonoid exposure. Overall, our findings provide a mechanistic basis for understanding inter-individual variability in flavonoid metabolism and its impact on personalized nutrition and health.

## 5. Conclusions

This study demonstrates the value of a prediction–validation approach in characterizing the gut microbial metabolism of dietary flavonoids and identifying the specific microorganisms and enzymes involved. We discovered that a CHS-like bacterial polyketide synthase can catalyze the C-ring cleavage of naringenin, highlighting a novel metabolic pathway in gut bacteria such as *Bacillus subtilis*. The metabolic products of this pathway, particularly 3,4-HPPA, exhibit distinct biological activities compared to the parent compound naringenin, specifically as ligands for AhR and NR4A1/2 receptors. These findings underscore the complex interplay between gut microbiota and dietary compounds in modulating host immune responses and suggest that individual variations in gut microbiota composition can significantly influence the metabolic fate and biological effects of dietary flavonoids. This work not only enhances our understanding of flavonoid metabolism in the gut but also provides a framework for future studies to explore the health implications of these microbial processes.

## 6. Limitations

We used a prediction scheme based on enzyme promiscuity to characterize gut bacterial flavonoid metabolism. At present, the predictions are limited to flavonoid compounds that have unique KEGG identifiers and corresponding chemical function format (KCF) files. Further, the metabolic model represents murine intestinal bacteria that we detected in anaerobic batch culture, cataloged in the miBC, and have annotated genomes. Thus, it is likely that the model does not capture the full repertoire of flavonoid metabolizing enzymes in murine gut microbiota. The anaerobic batch culture model used in the present study, while simple and reproducible, is a dynamic environment, where nutrient concentrations and bacterial abundance change over time. In this regard, future studies aimed at investigating the impact of flavonoid metabolism on microbiota community structure would benefit from the development of steady-state model systems.

## Figures and Tables

**Table 1:** Primer sequences used for real-time PCR to assess gene expression of human and mouse genes.

Gene	Primer Type	Sequence
***CYP1A1***	Sense	5′-GAC CAC AAC CAC CAA GAA C-3′
	Antisense	5′-AGC GAA GAA TAG GGA TGA AG-3′
***CYP1B1***	Sense	5′-GGA TAT CAG CCA CGA CGA AT-3′
	Antisense	5′-ATT ATC TGG GCA AAG CAA CG-3′
***UGT1A1***	Sense	5′-GAA TCA ACT GCC TTC ACC AAA AT-3′
	Antisense	5′-AGA GAA AAC CAC AAT TCC ATG TTC T-3′
***TBP***	Sense	5′-GAT CAG AAC AAC AGC CTG CC-3′
	Antisense	5′-TTC TGA ATA GGC TGT GGG GT-3′
**Mouse *Cyp1a1***	Sense	5′-CAG GAG AGC TGG CCC TTT A-3′
	Antisense	5′-TAA GCC TGC TC ATC CTG TG-3′
**Mouse *Cyp1b1***	Sense	5′-GGA TAT CAG CCA CGA CGA AT-3′
	Antisense	5′-ATT ATC TGG GCA AAG CAA CG-3′
**Mouse *Ugt1a1***	Sense	5′-ATG GCT TTC TTC TCC GGA AT-3′
	Antisense	5′-TCA GAA AAA GCC CCT ATC CC-3′
**Mouse *TBP***	Sense	5′-GAA CAA TCC AGA CTA GCA GCA-3′
	Antisense	5′-GGG AAC TTC ACA TCA CAG CTC-3′

## Data Availability

All data generated or analyzed during this study are included either in this article or in the Supplementary Materials.

## Supplementary Materials

The following supporting information can be downloaded at: https://www.mdpi.com/article/10.3390/metabo15030146/s1. Figure S1: Workflow for predicting microbial metabolites of dietary flavonoids using PROXIMAL. Figure S2: Naringenin biosynthesis pathway in plants. Table S1: PROXIMAL prediction results for gut microbial metabolism of selected flavonoids. Table S2: Distribution of predicted flavonoid metabolizing enzymes across different bacterial genera.
