# Supplementary material for: A Chalcone Synthase-like Bacterial Protein Catalyzes Heterocyclic C-Ring Cleavage of Naringenin to Alter Bioactivity Against Nuclear Receptors in Colonic Epithelial Cells
Source: Metabolites. 2025 Feb 21;15(3):146. doi: 10.3390/metabo15030146 (PMC11943482; doi:10.3390/metabo15030146)
Supplement: Supplementary file 1 [file metabolites-15-00146-s001.zip › Supplemental Information-Revised/Supplemental Information/Supplementary figures and table legends.docx]

**Supplementary Materials**

A Chalcone Synthase-Like Bacterial Protein Catalyzes Heterocyclic C-Ring Cleavage of Naringenin to Alter Bioactivity against Nuclear Receptors in Colonic Epithelial Cells

Ebru Ece Gülşan, Farrhin Nowshad, Meredith Davis Leigh, Jimmy W. Crott, Hyejin Park, Greg Martin, Stephen Safe, Robert S Chapkin, Arul Jayaraman, and Kyongbum Lee

**List of Supplemental Figures and Tables**

**Figure S1.** Workflow for predicting microbial metabolites of dietary flavonoids using PROXIMAL

**Figure S2.** Naringenin biosynthesis pathway in plants

**Table S1.** PROXIMAL prediction results for gut microbial metabolism of selected flavonoids. The products predicted by the operators for each of the 19 flavonoids (rows 7 – 35 in the spreadsheet) were used as substrates for a second round of predictions (rows 40 - 284). The column headings (in bold font) show the flavonoid names (rows 4 and 37) and KEGG COMPOUND numbers (C numbers, rows 5 and 38). The C numbers in each column represent a subset of the predicted flavonoid products that are cataloged in KEGG as metabolites.

**Table S2.** Distribution of predicted flavonoid metabolizing enzymes across different bacterial genera. The number of matches for a genus was normalized by the number of strains included in the model for the genus. Sheet 1: Each row is a different genus. Each column is a different combination of flavonoid and reaction type. Sheet 2: Each row is a different combination of genus and flavonoid. Each column is a different reaction type.

**Figure S1.** **Workflow for predicting microbial metabolites of dietary flavonoids using PROXIMAL.** (A) A previously assembled model of metabolic reactions for organisms detected in murine cecal culture [19] was pared down to a subset of representative strains that are also cataloged in Mouse Intestinal Bacterial Collection (miBC). These strains were searched against the KEGG GENOME and UnitProtKB databases to retrieve their enzymes and corresponding Enzyme Commission (EC) numbers. (B) Based on the RCLASS data associated with these EC numbers, atom group transformations in the enzymes' natural substrates were determined as previously described [31] and used to generate biotransformation operators. (C) If a flavonoid has an atom group that matches the natural substrate of an enzyme in the microbiota model, then the corresponding operator is applied to the flavonoid as a potential non-natural substrate, and one or more reaction products are predicted. (D) To mimic typical two-step cellular metabolism of exogenous chemicals, the products predicted by the operators for each of the 19 flavonoids were used as substrates for a second round of predictions.


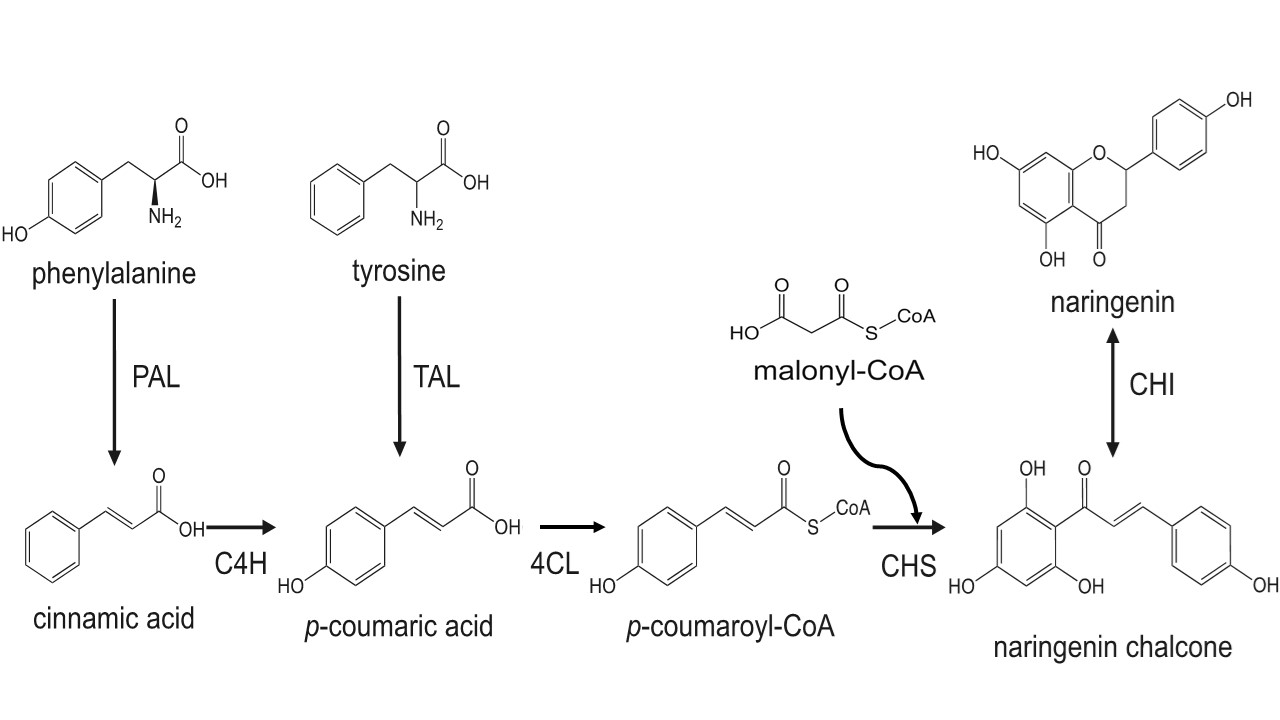


**Figure S2. Naringenin biosynthesis pathway in plants.** PAL: Phenylalanine ammonia-lyase, TAL: Tyrosine ammonia-lyase, C4H: Cinnamate-4-hydroxylase, 4CL: p-coumarate-CoA ligase, CHS: Chalcone synthase, CHI: Chalcone isomerase.
